# Supplementary figures and images for: Evaluation of an interprofessional follow-up intervention among people with type 2 diabetes in primary care—A randomized controlled trial with embedded qualitative interviews
Source: PLoS One. 2023 Nov 15;18(11):e0291255. doi: 10.1371/journal.pone.0291255 (PMC10650997; doi:10.1371/journal.pone.0291255)

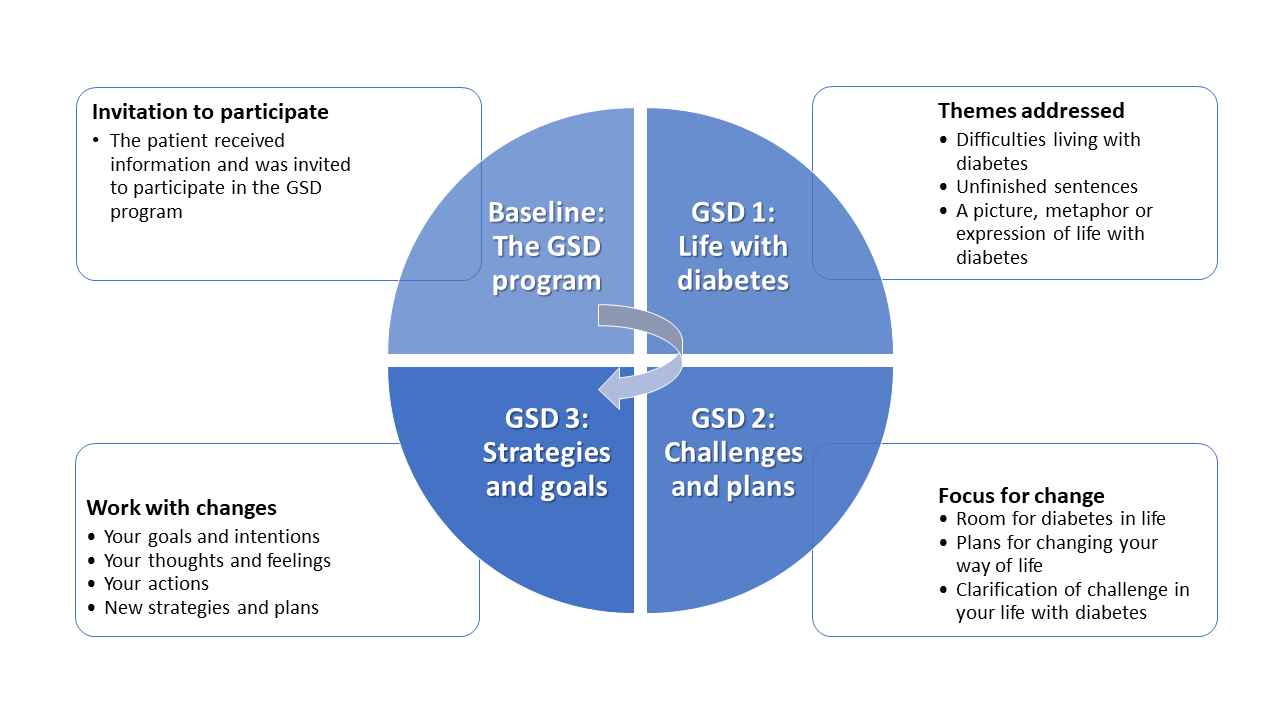

Supplement: S1 Fig — (TIF) [file pone.0291255.s002.tif]
