# Supplementary material for: Evaluation of an interprofessional follow-up intervention among people with type 2 diabetes in primary care—A randomized controlled trial with embedded qualitative interviews
Source: PLoS One. 2023 Nov 15;18(11):e0291255. doi: 10.1371/journal.pone.0291255 (PMC10650997; doi:10.1371/journal.pone.0291255)
Supplement: S1 File — (DOCX) [file pone.0291255.s003.docx]

**Study protocol for a randomised controlled trial:**

**Assessment of a team-based follow-up program for adults at risk of developing type 2 diabetes or with manifest type 2 diabetes in general practice**

Beate-Christin Hope Kolltveit^1,9^, Bjørg Frøysland Oftedal^2^, Anne Haugstvedt^1^, Arun K Sigurdardottir^3,4^, Jannicke Igland^1,5^, Vibeke Zoffmann^6,7^, Anne Karen Jenum^8^, Vigleik Kolltveit^9^, David Richards^1,10^, Marit Graue^1^

^1^Department of Health and Caring Sciences, Western Norway University of Applied Sciences, Bergen, Norway; ^2^Faculty of Health Sciences, University of Stavanger, Stavanger, Norway

^3^School of Health Sciences, University of Akureyri, Akureyri, Iceland

^4^Akureyri Hospital, Akureyri, Iceland; ^5^Department of Global Health and Primary Health Care, University of Bergen, Bergen, Norway; ^6^Research unit for Women`s and Children`s Health, Julie Marie Centre, Rigshospitalet, Copenhagen, Denmark; ^7^Institute of Public Health Copenhagen University, Copenhagen, Denmark

^8^General Practice Research Unit (AFE), Department of General Practice, University of Oslo, Institute of Health and Society, Norway

^9^Vossevangen medical centre, Voss, Norway ^10^Institute for Health Research, College of Medicine and Health, University of Exeter, Exeter, United Kingdom

**Abstract**

**Introduction** High prevalence of non-communicable diseases is a worldwide public health challenge resulting in morbidity and mortality, as well as significant hospitalization rates and huge personal and societal costs. Several trials in the last decades have demonstrated that preventive strategies (i.e. lifestyle modification) can delay or prevent the development of Type 2 diabetes Mellitus (T2DM) in high-risk individuals. We present the protocol for a randomised controlled trial to assess the effectiveness of a theory-driven Guided Self-Determination (GSD) intervention designed to improve health-care services, prevent chronic diseases, and deliver better risk-factor management among people at risk of developing T2DM or with manifest T2DM.

**Methods and analysis** We will recruit 308 participants from general practice into an intervention involving general practitioners (GPs) and registered nurses using GSD versus standard care GP consultation only. The primary outcome will be changes in the Patient Activation Measure (PAM-13). As secondary outcome measures, we will evaluate the effect of the intervention on BMI, waist circumference, HbA1c, Problem Areas in Diabetes (PAID-5) scale, WHO Well-Being Index (WHO-5), Quality of life (QOL Health Item - 2 items), EQ-5D, HLS-EU-Q12 and Perceived competence diabetes scale (PCDS – 4 items). The effect of the intervention will be measured after 3, 6 months and 12 months and estimated using linear mixed effects regression for continuous outcomes and mixed effects logistic regression and generalized estimating equations for binary outcomes. We will conduct qualitative interviews among patients, health care professionals and user representatives to further inform the evaluation of the program. We will analyse the qualitative data by using thematic analysis. In addition, we will examine intervention fidelity.

**Ethics and** **dissemination** Ethical approval has been obtained from the South-Eastern Norway Regional Committee for Medical and Health Research Ethics (2019/28/REK sør-øst A). The trial is registered in ClinicalTrials.gov (ID: NCT04076384).

**Strengths and limitations of this study**

- This study has the potential to extend the knowledge of the effects of delegated independent tasks and nurses’ roles in a team-based approach in general practice on identifying need for follow-up.
- The study will facilitate adequate self-management support by using a theory-driven Guided-Self-Determination intervention in the follow-up of people with high-risk of or with manifest type 2 diabetes mellitus.
- The study will embed qualitative interviews with patients, healthcare professionals and user representatives to gain insight into their experience when participating in the study.
- A limitation is that the assignment of general practices is limited to practices with trained nurses in Guided-Self-Determination that can use the team-based person-centred intervention as support for their nursing follow-up consultations.
- We will not evaluate long-term effects (i.e. >12 months).

**INTRODUCTION**

High prevalence of non-communicable diseases is a worldwide public health challenge resulting in morbidity, and mortality, as well as significant hospitalization rates and huge personal and societal costs. Among chronic conditions, diabetes prevalence ranks very high, expected to reach 592-642 million people by 2035-2040 ^1,2^. One in ten adults is estimated to have diabetes by 2040. Type 2 diabetes (T2DM) is associated with various and serious complications such as cardiovascular disease and kidney disease ^2^. Together with risk factors such as high blood pressure and tobacco use, high blood glucose has been identified as the third largest risk factor for diabetes related mortality globally ^2^. It is estimated that 1 in 12 of all-cause adult deaths globally are attributable to diabetes ^3^. Being overweight, obese, or having an unhealthy lifestyle are strongly linked to T2DM regardless of genetic risk ^4^. Thus, a major concern for public health is to halt the rise in obesity in the general population to reduce the risk of developing diabetes ^5^. In 2014, the most recent year for which global estimates are available, more than one in three adults over 18 years of age were overweight and more than one in ten were obese ^6^. To prevent obesity and to delay serious complications among people with manifest T2DM, adequate primary care services are essential. Several trials have demonstrated that lifestyle modifications can prevent or delay the development of T2DM in high-risk individuals ^2,7,8^. Lifestyle intervention aiming at weight reduction, a healthy diet and increased physical activity in high-risk individuals has a long-lasting effect in the prevention of T2DM ^8,9^. Moreover, lifestyle interventions can prevent or delay further development among people with manifest T2DM. The DIRECT study found that an intensive lifestyle follow-up program in the early stages of T2DM, supported by registered nurses and dieticians in general practice led to diabetes remission in 46% of participants in the intervention group compared to 4% in the control group ^7^.

Assessing people’s skills and knowledge is essential in a person-centred and individual approach to problem solving for people at risk of chronic disease or with manifest chronic disease. Interventions should focus on these aspects ^10^. To assess people`s ability to engage with their own health behaviour might be measured by using a patient activation measurement instrument (PAM) ^11^. The degree of activation and engagement might contribute to better self-management and overall better health outcomes ^12,13^.

A recent Norwegian study found that intensive individual-level intervention seems to be an important strategy in preventing T2DM ^14^. General practitioners (GPs) in Norway are in general overloaded with limited possibilities for more intensive follow-up of people at risk of or with chronic disease ^15^. The increasing workload in general practices is also reported in other countries ^16^. Further research to identify feasible strategies for the implementation of healthy lifestyle interventions for people at risk of or with manifest T2DM is important. Involvement of nurses in education and counselling of patients has shown to have positive effects on hospital admissions and mortality ^17^. Thus, team-based follow-up in primary care is a promising strategy to improve quality outcomes in a strengthened primary care system. However, a call for further studies on the use of multi-professional and team-based follow-up in Norwegian primary care is warranted ^18^. Especially, there is limited research on delegated independent tasks to nurses in general practice in Norway. Such studies conducted in the Norwegian context are important because nurses traditionally have had a less autonomous role in the care and follow-up of persons in general practice suffering from common chronic diseases such as diabetes compared to other Scandinavian countries ^19^.

The potential for improvements in patients’ attentiveness to medical treatment and better adherence to lifestyle change is unambiguous. A systematic literature review suggested that person-centred care may lead to significant improvements for the patients, but the implementation and relevant effects needs to be assessed in more studies ^20^. This study strives to improve the quality in health-care services in general practice by applying the team-based Guided Self-Determination intervention. Guided Self-Determination is a theory-driven counselling approach with the purpose to guide people and health care professionals through mutual reflection and activation ^21,22^.

We aimed to develop, test and evaluate a team-based Guided Self-Determination (GSD) intervention in general practice designed to improve patient activation and self-management among people with T2DM or at risk of developing T2DM. The effect of the intervention will be compared to standard care (GP consultation only) in terms of patient activation and self-management.

**METHODS**

We will undertake a randomised controlled trial (RCT) among people at risk of developing T2DM or with manifest T2DM to test the effect of the intervention using guidance from the Medical Research Council (MRC) framework for developing, testing and evaluating the GSD intervention as a team-based person-centred approach in general practice ^23^. We report our protocol using Standard Protocol Items: Recommendations for Interventional Trials (SPIRIT) checklist <https://www.spirit-statement.org/> ^24,25^.

**Trial population, eligibility criteria and recruitment**

We will identify people with increased risk of developing T2DM (by the Finnish Diabetes Risk Calculator ^26,27^ (FINDRISC) ≥ 15 or BMI ≥30) and those with manifest T2DM (by HbA1c ≥48 mmol/L) aged between 20-80 years to participate in the study. We will exclude people with severe somatic disease (i.e. severe cancer, severe heart failure, end stage renal disease), severe psychiatric diagnosis (e.g. severe depression, bipolar disorder, schizophrenia), or recorded cognitive deficiency (e.g. Down’s syndrome, Alzheimer’s disorder) or who do not write, speak or understand Norwegian. Separate trials will be performed for people with risk of developing T2DM and people with manifest T2DM.

*Sample size*

We performed power analysis based on the 13-items Patient Activation Measure (PAM-13) as the primary outcome (patients’ ability for self-management of one’s health or chronic condition) with a total score ranges from 0-100 (best) ^11^. Based on a previous study we expect a mean (SD) PAM-13 score at baseline of approximately 52 (14) ^11^. For either one of the participants at risk of developing T2DM or manifest T2DM, we estimated the required sample size to be 64 participants to detect a treatment effect of 7 points (0.5 SD) with 80% power and a two-sided 0.05 significance level. To account for possible dropouts, we will increase the sample to 77 (20% increase) in each group. Accordingly, we will need to include a total of 154 patients in each group (among people with risk of T2DM and with manifest T2DM), e.g n= 77 in the intervention with GSD and n= 77 in the GP follow-up only. We will recruit participants through a screening questionnaire handed out to persons in the waiting room at GP offices. The screening questionnaire will include questions about manifest diabetes in addition to the FINDRISC. According to Jølle et. al ^14,28^ the prevalence of elevated FINDRISC (≥15) among 47,694 adults in the third survey of the Nord-Trøndelag Health Study (HUNT3, 2006–08) was 11%. Thus, to identify at least 154 patients at high-risk of developing T2DM from a population with an estimated prevalence of elevated FINDRISC score of 11%, our screening population in general practice will need to include at least 1400 people (154/0.11). To include enough persons with manifest T2DM we will also identify people with T2DM through the medical records at the GP office and ask them if they want to participate.

**Adapting the intervention for use in general practice for people in risk of developing T2DM or with manifest T2DM**

We have previously developed and adapted routines for using GSD in the team-based person-centred follow-up program in general practice, and conducted feasibility studies to explore benefits and challenges using the person-centred GSD program among adults in different settings and formats ^29-33^. However, further adaption was needed to refine the program for use in a team-based follow-up program in a busy general practice context. Also, we adapted the intervention to include people at risk of developing T2DM. Through this adaptation process we learned that it was beneficial to reduce the total number of semi structured reflection sheets from the original program that we have used in a previous web-based intervention study ^30,31^. Thus, the intervention now consists of a stepwise approach with 1-4 consultations over 12 months (Fig. 1) and a more flexible use of reflection sheets considering the condition as well as each individual’s needs to reflect upon lifestyle modifications and problem-solving communication. To maintain quality in the use of GSD the nurses take part in a training program for advanced communication skills from the GSD program, life skills and self-management, supervised exercises, and a reading list.

*Figure 1 Overview of reflection sheets*

We will test the feasibility of the adapted intervention in one GP practice in Vestland county with ≥3 GPs and ≥1 nurse(s). Trained nurses and GPs will assess the patients’ individual risk profiles with scores over target level and refer a total of 10-15 patients to participate. GSD is a theory‐driven counselling approach ^22,34,35^. Originally, the GSD program comprised seven face-to-face consultations with 21 semi structured reflection sheets for people with type 1 diabetes. Reflection sheets and communication skills (mirroring, active listening and values clarification response) aim to guide patients and nurses through a mutual reflection, empowering the patient to become self-determined, achieve life skills and enhancing their self-management skills in living with a chronic disease. In the present study, trained GSD nurses in the intervention group will also involve the GPs in the team-based consultations.

The intervention will mainly be face-to-face consultations, however also telephone or video consultations might be considered if suitable in the follow-up. Between the consultations, the participants will fill in a set of reflection sheets using their own words and drawings to express and reflect on their experiences and difficulties with the management of their disease in daily life as well as to formulate behavioural goals and plans to achieve improved self-management. The nurses using GSD will also capture this in the structured consultations as well as report on this in the medical records after the consultations. Structured training by telephone, video or face-to-face meetings for the nurses is provided before the intervention starts as well as every month to ensure that the intervention is conducted according to protocol.

*Control:* The patients in the control group will receive usual care, follow-up with GP consultations only (without the use of GSD).

*Patient recruitment:*

We will conduct the study in four GP practices in Vestland and Viken counties with ≥3 GPs and ≥1 nurse(s). We will hand out screening questionnaires to people in the waiting room at the four GP practices. Only people who are listed as patients to the specific GPs` practices will be asked to answer the questionnaires.

*Data and methods:* We will randomise the patients into an intervention arm with team-based person-centred structured consultations (with a nurse using GSD to identify psychosocial and behavioural problems and improving patient-provider communication) versus standard care (GP consultation only) by computer generated numbers. The randomization sequences will be generated by an independent person using statistical software. We will begin enrolment of the first patient in the follow-up program in December 2019 and continue with participant recruitment for the intervention until baseline data collection have been fulfilled at all GP practices. The intervention is designed to evaluate a change in patient activation and self-management, in addition we will train the nurses in GSD, therefore blinding of the intervention is not possible. An internal pilot is carried out on the first ten patients entering the study ^36,37^. These patients will be included in the final analysis if appropriate. We will use the diabetes form in the electronic patient records for those with manifest T2DM to extract demographic and clinical data as well as questionnaire data to assess the effect of the intervention after three, six and twelve months.

*Primary outcome (table 1):* Patient Activation Measure (PAM-13). The PAM-13 instrument ^38,39^ capture patient's knowledge, skills, and confidence for self-management living with chronic conditions. It has 13 items with four response categories with scores from 1 to 4: “strongly disagree” (1), “disagree” (2), “agree” (3) and “agree strongly” (4). The item scores are transformed to a 0 to 100 scale (0 = lowest activation level, 100 = highest activation level). The manual suggests cut-off points to categorize patients into four levels of activation reflecting their engagement in health behaviour. Level 1 (0 - 47) indicates that the individual may not believe the patient role is important, level 2 (47.1 - 55.1) indicates that a patient lacks confidence and knowledge to take action, level 3 (55.2 - 67.0) indicates that a patient has started to engage in the recommended health behaviour, and level 4 (67.1 - 100) indicates that a patient is proactive concerning health and engages in many types of recommended health behaviour. PAM-13 has been translated to Norwegian, validated, and used in previous Norwegian studies ^11,40^.

*Secondary outcomes (table 1)*: BMI, waist circumference, HbA1c, Problem Areas in Diabetes (PAID-5), WHO Well-Being Index (WHO-5), Quality of life (QOL Health Item - 2 items), EQ-5D, HLS-EU-Q12 and Perceived competence diabetes scale (PCDS – 4 items). Moreover, we will record hospitalization events and number of GP’s consultations from the electronic patient records after the intervention is ended.

**Table 1.** Outcome measures.

| **Chronic disease** | **Primary outcome** | **Secondary outcomes** |
| --- | --- | --- |
| Risk of T2DM | Patient Activation Measure (PAM 13) – reduction of 5-10% | Weight reduction  Waist circumference reduction  Blood pressure reduction  Lifestyle adherence: physical activity >30 min/day  WHO Well-Being Index (WHO-5)  Quality of life (QOL Health Item - 2 item)  EQ-5D  HLS-EU-Q12 |
| Manifest T2DM | Patient Activation Measure (PAM 13) – reduction of 5-10% | Weight reduction  HbA1c reduction  Waist circumference reduction  Lifestyle adherence: physical activity >30 min/day  Blood pressure reduction  Adherence to medical treatment  Problem Areas in Diabetes (PAID-5)  WHO Well-Being Index (WHO-5)  Quality of life (QOL Health Item - 2 item)  EQ-5D, HLS-EU-Q12  Perceived competence diabetes scale (PCDS– 4 items)  We will record hospitalization events and number of GP’s consultations from the electronic diabetes form. |

*Statistical analysis:* The effect of the intervention will be measured after 3, 6 months and 12 months as difference in change in mean values of the outcomes. The effect will be estimated using linear mixed effects regression models and reported as regression coefficients with 95% confidence intervals for continuous outcomes. For binary outcomes we will use mixed effects logistic regression and report the effect as odds ratios (OR) with 95% confidence intervals. In case of differential drop-out we will perform sensitivity analyses with adjustment for characteristics which differ between those who complete the follow-up and those who are lost to follow-up. If follow-up data for binary outcomes seem to be missing completely at random we will also apply generalized estimating equations (GEE) with log-link to estimate risk ratios (RR). We will use two-sided tests with 5% as the significance level for all models.

**Conducting qualitative interviews**

The participating patients, nurses, GPs, and user representatives will be invited to share their experiences implementing the team-based person-centred GSD follow-up program.

The qualitative study comprises individual interviews with 20 patients (risk of T2DM (n=10) T2DM (n=10)), and 12 individual interviews with nurses (n=6) and GPs (n=6) from the four GP practices in Vestland and Viken counties participating in the RCT study. We will use a semi-structured interview guide. Included topics are barriers to and facilitators of implementing the intervention, the feasibility of study procedures, the training they received, and perceived effectiveness of using the GSD tools in regular consultations. Moreover, we will conduct interviews with all the user representatives throughout the evaluation of study (n=4). It is important to provide insight into health service users’ perspectives, in terms of patient and public involvement in designing projects, shaping the research processes, and informing the outcomes of the trial.

We will transcribe the interviews and analyse them using thematic analysis as described by Braun and Clarke ^41,42^. First, we will read the data several times for the researcher to become familiar with the data. Then, the data needs to be initially coded, so that the material can be further organized by potential extracts relevant to each code in phase three. Subsequently, the topics are re-organized and new codes created for data falling outside the themes. Then, the themes are further determined and labelled, meaningful patterns identified, and finally outlined and agreed upon by all authors.

**Examining the fidelity of applying the GSD intervention**

We will examine intervention fidelity among nurses applying GSD in the consultations. In addition, we will examine the quality of and adherence to using GSD by reflection notes from the nurses after their consultations with the patients. After three months of implementing the intervention, the nurses make notes for every fifth patient consultation on how they experience their use of the GSD in the consultation. We have developed a case report form for collecting these data. This report form consists of key elements from the GSD method on how to deliver the intervention as it is designed. In addition, the nurses will complete a self-report questionnaire to report the duration of each consultation, whether the time available was sufficient or not, along with an evaluation of their own perception of their capability to deliver the intervention as designed. They will also report on their motivation to apply the intervention integrated into part of routine care. Descriptively analysis of these report forms will be conducted to examine if the GSD intervention consultations were suitable and conducted in accordance with the given procedures.

**PATIENT AND PUBLIC INVOLVEMENT (PPI)**

Two user representatives of patients will participate to ensure that patients’ needs and concerns are met ^43^. In addition, four user representatives (nurses and GP’s) from general practice representing both genders, different ages, and varying years of professional experience will contribute to all phases of the project. Firstly, the user groups have contributed to discussing the relevance of the study objectives and justification for undertaking the research. Secondly, they have contributed to decisions on methods related to data collection, measurements, and tools for risk identification. Thirdly, the user groups have influenced our developing recruitment methods, eligibility criteria and strategies for achieving adequate participant enrolment. Moreover, they have participated in the development of adequate written patient information. In the evaluation phase, they will contribute to identifying and refining delivery mode and dose (intensity of team-based follow-up), which are features that will make the application realistic and user friendly. We will report PPI by using the GRIPP2 – SF guidelines ^44^ to ensure that the follow-up of user representatives is achieved to a high standard.

**ETHICS AND DISSEMINATION**

The study has obtained ethical approval from the South-Eastern Norway Regional Committee for Medical and Health Research Ethics (2019/28/REK sør-øst A). The project will be carried out in accordance with the Helsinki Declaration and designed and reported in accordance with the CONSORT guidelines (Consolidated Standards of Reporting Trials). Further information can be obtained from ClinicalTrials.gov (ID: NCT04076384). The Western Norway University of Applied Sciences is the responsible research institution where we will store study data on a secure research server. To protect the participants confidentiality, the names of the potential participants and those enrolled will be stored separately. The principal investigator and some clearly identified members of the project group will have access to the data. If some important protocol modifications occur, we will communicate this to the ethics committee and ClinicalTrials.gov. To ensure good medical quality in the follow-up of the participants the responsible GPs reads all notes from the nurses in the patient`s medical record. Along with input from the user groups, we will publish the results in peer reviewed scientific journals, popular scientific journals and social media platforms, at international research conferences, national seminars and meetings for patients and clinicians.

**DISCUSSION**

We have developed a team-based person-centered GSD intervention in general practice designed to improve patient activation and self-management among people with T2DM or at risk of developing T2DM. The effect of the intervention will be evaluated in separate RCT-studies in the two patient groups. The study will provide knowledge to working team-based with using the theory-driven GSD approach in the consultations with people at risk of diabetes or with manifest disease in general practice. This study can also provide us with knowledge on using an adapted GSD program in a busy general practice to enhance self-management. Inadequate services and follow-up of people with chronic conditions may lead to a range of complications and adverse events. Insufficient self-management support and guidance give rise to poor patient adherence to medical treatment and lifestyle advice, which may be avoided by more coordinated team-based person-centred approaches. We have designed the intervention for improving patients’ ability for self-management of their health and/or chronic condition and we use a Patient Activation Measure (PAM-13) as the primary outcome in this trial. Supporting patient activation is found to be a potential pathway to help achieve a goal comprising health outcome such as a more healthy behaviour ^45^.

Moreover, we will in this study gain insights for factors potentially improving health-care services, extend the knowledge of delegated independent tasks and nurses’ roles in the prevention of chronic diseases as well as in better risk-factor management in general practice. The importance of early, comprehensive lifestyle change as the primary target of a T2DM prevention strategy is shown ^7^.

**Acknowledgements**

The authors would like to thank the patients, user representatives involved and the GPs and nurses working in general practice for their contribution to the study protocol. Thanks to the Norwegian Nurses Association and the Western Norway University of Applied Sciences.

**Contributors**

BCHK and MG applied for funding for this study. BCHK, MG, AKS and DR developed the study design with involvement of JI, AH, BFO, VZ, AKJ, KB and VK. BCHK and MG drafted the protocol. VZ developed the GSD method. All authors edited and critically reviewed the manuscript and read and approved the final version.

**Funding**

The Norwegian Nurse Association has reviewed the application and provided a postdoctoral position to further develop the project, and Western Norway University of Applied Sciences contributed with faculty research resources and running funds. Grant number is not applicable.

**Competing interests**

None declared.

**Patient consent**

All patients recruited will fill out written informed consent form prior to participation.

**References**

1. Guariguata L, Whiting DR, Hambleton I, et al. Global estimates of diabetes prevalence for 2013 and projections for 2035. *Diabetes research and clinical practice* 2014;103(2):137-49.

2. Cefalu WT, Buse JB, Tuomilehto J, et al. Update and next steps for real-world translation of interventions for type 2 diabetes prevention: reflections from a diabetes care editors’ expert forum: Am Diabetes Assoc, 2016.

3. IDF DAG. Update of mortality attributable to diabetes for the IDF Diabetes Atlas: Estimates for the year 2013. *Diabetes research and clinical practice* 2015;109(3):461.

4. Obesity and unfavourable lifestyle increase type 2 diabetes-risk independent of genetic predisposition: a retrospective population-based cohort study. 55th Annual Meeting of the European Association for the Study of Diabetes (EASD); 2019.

5. Swinburn BA, Sacks G, Hall KD, et al. The global obesity pandemic: shaped by global drivers and local environments. *The Lancet* 2011;378(9793):804-14.

6. WHO. Diabetes mellitus: WHO; 2018 [Available from: <http://www.who.int/mediacentre/factsheets/fs312/en/>

7. Lean ME, Leslie WS, Barnes AC, et al. Primary care-led weight management for remission of type 2 diabetes (DiRECT): an open-label, cluster-randomised trial. *The Lancet* 2018;391(10120):541-51.

8. Lean ME, Leslie WS, Barnes AC, et al. Durability of a primary care-led weight-management intervention for remission of type 2 diabetes: 2-year results of the DiRECT open-label, cluster-randomised trial. *The Lancet Diabetes & Endocrinology* 2019;7(5):344-55.

9. Lindström J, Peltonen M, Eriksson J, et al. Improved lifestyle and decreased diabetes risk over 13 years: long-term follow-up of the randomised Finnish Diabetes Prevention Study (DPS). *Diabetologia* 2013;56(2):284-93.

10. King DK, Glasgow RE, Toobert DJ, et al. Self-efficacy, problem solving, and social-environmental support are associated with diabetes self-management behaviors. *Diabetes care* 2010;33(4):751-53. doi: 10.2337/dc09-1746

11. Moljord IEO, Lara-Cabrera ML, Perestelo-Perez L, et al. Psychometric properties of the Patient Activation Measure-13 among out-patients waiting for mental health treatment: A validation study in Norway. *Patient education and counseling* 2015;98(11):1410-17.

12. Hibbard JH, Greene J. What the evidence shows about patient activation: better health outcomes and care experiences; fewer data on costs. *Health affairs* 2013;32(2):207-14.

13. Donald M, Ware RS, Ozolins IZ, et al. The role of patient activation in frequent attendance at primary care: a population-based study of people with chronic disease. *Patient education and counseling* 2011;83(2):217-21.

14. Jølle A, Åsvold BO, Holmen J, et al. Basic lifestyle advice to individuals at high risk of type 2 diabetes: a 2-year population-based diabetes prevention study. The DE-PLAN intervention in the HUNT Study, Norway. *BMJ Open Diabetes Research and Care* 2018;6(1):e000509.

15. Nilsen L. Dagens Medisin: <https://www.dagensmedisin.no>; 2018 [Available from: <https://www.dagensmedisin.no/artikler/2018/04/10/6-av-10-fastleger-har-vurdert-a-slutte-pa-grunn-av-arbeidspress>

16. Moth G, Vestergaard M, Vedsted P. Chronic care management in Danish general practice - a cross‒sectional study of workload and multimorbidity. *BMC Family Practice* 2012;13(1):52. doi: 10.1186/1471-2296-13-52

17. Martínez-González NA, Djalali S, Tandjung R, et al. Substitution of physicians by nurses in primary care: a systematic review and meta-analysis. *BMC Health Services Research* 2014;14(1):214. doi: 10.1186/1472-6963-14-214

18. Sørensen M, Stenberg U, Garnweidner-Holme L. A scoping review of facilitators of multi-professional collaboration in primary care. *International journal of integrated care* 2018;18(3) doi: 10.5334/ijic.3959

19. Iversen T, Anell A, Häkkinen U, et al. Coordination of health care in the Nordic countries. *Nordic Journal of Health Economics* 2016;4(1):pp. 41-55.

20. Olsson LE, Jakobsson Ung E, Swedberg K, et al. Efficacy of person‐centred care as an intervention in controlled trials–a systematic review. *Journal of clinical nursing* 2013;22(3-4):456-65.

21. Zoffmann V, Kirkevold M. Life versus disease in difficult diabetes care: conflicting perspectives disempower patients and professionals in problem solving. *Qualitative Health Research* 2005;15(6):750-65.

22. Zoffmann V, Kirkevold M. Relationships and their potential for change developed in difficult type 1 diabetes. *Qualitative Health Research* 2007;17(5):625-38.

23. Craig P, Dieppe P, Macintyre S, et al. Developing and evaluating complex interventions: the new Medical Research Council guidance. *International journal of nursing studies* 2013;50(5):587-92.

24. Chan A-W, Tetzlaff JM, Altman DG, et al. SPIRIT 2013 Statement: Defining Standard Protocol Items for Clinical Trials. *Annals of Internal Medicine* 2013;158(3):200-07. doi: 10.7326/0003-4819-158-3-201302050-00583

25. Chan A-W, Tetzlaff JM, Gøtzsche PC, et al. SPIRIT 2013 explanation and elaboration: guidance for protocols of clinical trials. *BMJ : British Medical Journal* 2013;346:e7586. doi: 10.1136/bmj.e7586

26. Tankova T, Chakarova N, Atanassova I, et al. Evaluation of the Finnish Diabetes Risk Score as a screening tool for impaired fasting glucose, impaired glucose tolerance and undetected diabetes. *Diabetes research and clinical practice* 2011;92(1):46-52. doi: 10.1016/j.diabres.2010.12.020

27. Lindström J, Peltonen M, Eriksson JG, et al. Determinants for the effectiveness of lifestyle intervention in the Finnish Diabetes Prevention Study. *Diabetes care* 2008;31(5):857-62. doi: 10.2337/dc07-2162

28. Jølle A, Midthjell K, Holmen J, et al. Impact of sex and age on the performance of FINDRISC: the HUNT Study in Norway. *BMJ Open Diabetes Research &amp; Care* 2016;4(1):e000217. doi: 10.1136/bmjdrc-2016-000217

29. Karlsen B, Oftedal B, Lie SS, et al. Assessment of a web-based Guided Self-Determination intervention for adults with type 2 diabetes in general practice: a study protocol. *BMJ open* 2016;6(12):e013026.

30. Lie SS, Karlsen B, Niemiec CP, et al. Written reflection in an eHealth intervention for adults with type 2 diabetes mellitus: a qualitative study. *Patient preference and adherence* 2018;12:311.

31. Lie SS. An eHealth intervention based on the Guided Self-determination program for adults with type 2 diabetes in general practice, 2018.

32. Mohn J, Graue M, Assmus J, et al. The effect of guided self-determination on self-management in persons with type 1 diabetes mellitus and HbA1c≥ 64 mmol/mol: a group-based randomised controlled trial. *BMJ open* 2017;7(6)

33. Karlsen B, Rasmussen Bruun B, Oftedal B. New possibilities in life with type 2 diabetes: experiences from participating in a guided self-determination Programme in general practice. *Nursing research and practice* 2018;2018

34. Zoffmann V, Kirkevold M. Realizing Empowerment in Difficult Diabetes Care A Guided Self-Determination Intervention. *Qualitative health research* 2012;22(1):103-18.

35. Zoffmann V, Lauritzen T. Guided self-determination improves life skills with type 1 diabetes and A1C in randomized controlled trial. *Patient education and counseling* 2006;64(1):78-86.

36. Lancaster GA, Dodd S, Williamson PR. Design and analysis of pilot studies: recommendations for good practice. *Journal of evaluation in clinical practice* 2004;10(2):307-12. doi: 10.1111/j..2002.384.doc.x

37. Richards DA, Hallberg IR. Complex interventions in health: an overview of research methods. New York: Routledge 2015:121-82.

38. Hibbard JH, Stockard J, Mahoney ER, et al. Development of the Patient Activation Measure (PAM): conceptualizing and measuring activation in patients and consumers. *Health services research* 2004;39(4p1):1005-26.

39. Hibbard JH, Mahoney ER, Stockard J, et al. Development and testing of a short form of the patient activation measure. *Health services research* 2005;40(6p1):1918-30.

40. Steinsbekk A. Patient activation measure. *Tidsskrift for den Norske laegeforening: tidsskrift for praktisk medicin, ny raekke* 2008;128(20):2316-18.

41. Braun V, Clarke V. Using thematic analysis in psychology. *Qualitative research in psychology* 2006;3(2):77-101.

42. Braun V, Clarke V. Successful qualitative research: A practical guide for beginners: sage 2013.

43. Harris J, Graue M, Dunning T, et al. Involving people with diabetes and the wider community in diabetes research: a realist review protocol. *Systematic reviews* 2015;4(1):146.

44. Staniszewska S, Brett J, Simera I, et al. GRIPP2 reporting checklists: tools to improve reporting of patient and public involvement in research. *Research involvement and engagement* 2017;3(1):13.

45. Greene J, Hibbard JH, Sacks R, et al. When patient activation levels change, health outcomes and costs change, too. *Health Affairs* 2015;34(3):431-37.
