# Supplementary material for: Evaluation of an interprofessional follow-up intervention among people with type 2 diabetes in primary care—A randomized controlled trial with embedded qualitative interviews
Source: PLoS One. 2023 Nov 15;18(11):e0291255. doi: 10.1371/journal.pone.0291255 (PMC10650997; doi:10.1371/journal.pone.0291255)
Supplement: S2 File — (DOCX) [file pone.0291255.s004.docx]

**SI Table 1.** Standardised medical report produced by the interprofessional team.

| Standardised components available in the medical report to ensure that the following were monitored to satisfy requirements in clinical guidelines.   - Review of the medications used by the patient. - Review of possible side effects of medications. - Review of interprofessional follow-up (ophthalmologist, cardiologist). - Review of the blood tests. - Blood pressure. - Height, weight, waist measurement. - Review of the patient`s activity in daily life. - Review of the patient`s dietary habits. - Review of the cardiovascular risk factors. - Electrocardiogram result in the text. - A structured follow-up of the feet: visual check of the foot, check for the pulse in arteria dorsalis pedis and arteria tibialis posterior, monofilament test. |
| --- |
| Principles available in the medical report to ensure fidelity implying the GSD approach.   - At present, what do you find difficult living with the condition? - Expression of life with diabetes by unfinished sentences, pictures, and metaphors. - Room for the condition in your life. - Your plans for changing your way of life. - Clarification of challenge in your life with the condition. - Goals and intentions. - Your thoughts and feelings. - Your actions. - New strategies and plans. |

**SI Table 2.** Interview guide.

| **Overall themes** |
| --- |
| What is your experience with participating in this study? |
| How did you experience the meetings with the nurse and the primary care physician regarding your health condition? |
| What about the follow-up when participating in the study? Any changes? |
| Did you experience something negative? Did you experience something positive? |
| How did you experience the counselling from the nurse or the doctor? Is there anything that could have been different? |
| What is your experience with interprofessional follow-up? |
| Were there any changes in your health condition during the last year? |
| Have you experienced changes in self-management during this time? |

**SI Table 3**. Within-group changes in outcome measures.

|  |  | Mean change from baseline | | | | | |
| --- | --- | --- | --- | --- | --- | --- | --- |
|  | Baseline | 3 months | | 6 months | | 12 months | |
|  | Mean (SD) | B (95% CI) | p-value | B (95% CI) | p-value | B (95% CI) | p-value |
| Primary outcome |  |  |  |  |  |  |  |
| PAM-13 |  |  |  |  |  |  |  |
| Control | 68.3 (12.8) | 6.2 (2.0,10.5) | 0.004 | 3.0 (-1.3,7.4) | 0.17 | 5.2 (0.7,9.8) | 0.03 |
| Intervention | 76.3 (13.4) | 0.7 (-3.3,4.7) | 0.73 | 1.9 (-2.2, 5.9) | 0.37 | -2.0 (-6.6, 2.7) | 0.41 |
| Secondary outcomes |  |  |  |  |  |  |  |
| BMI (kg/m^2^) |  |  |  |  |  |  |  |
| Control | 30.3 (4.5) | 0.1 (-0.2, 0.5) | 0.45 | -0.2 (-0.6, 0.2) | 0.46 | -0.5 (-1.1, 0.2) | 0.15 |
| Intervention | 29.1 (4.6) | -0.2 (-0.7, 0.3) | 0.42 | -0.1 (-0.6, 0.4) | 0.76 | -0.6 (-1.2, 0.0) | 0.045 |
| Weight (kg) |  |  |  |  |  |  |  |
| Control | 92.4 (17.7) | 0.0 (-1.5, 1.5) | 0.99 | -0.6 (-2.6, 1.3) | 0.53 | -0.5 (-3.7, 2.6) | 0.75 |
| Intervention | 87.2 (15.9) | 0.2 (-1.2, 1.6) | 0.82 | -0.9 (-2.3, 0.6) | 0.23 | -1.8 (-3.3, -0.3) | 0.02 |
| Waist circumference (cm) |  |  |  |  |  |  |  |
| Control | 107.9 (12.6) | 0.4 (-1.4, 2.1) | 0.68 | 0.5 (-1.3, 2.3) | 0.58 | -1.0 (-2.9, 1.0) | 0.34 |
| Intervention | 106.2 (11.5) | -2.4 (-5.8, 1.1) | 0.18 | -0.4 (-3.7, 3.0) | 0.84 | -3.9 (-7.3, -0.6) | 0.02 |
| HbA1c (mmol/mol) |  |  |  |  |  |  |  |
| Control | 51.9 (7.8) | 2.7 (-4.7, 10.0) | 0.48 | 1.4 (-7.7, 10.5) | 0.76 | 7.7 (-6.2, 21.6) | 0.28 |
| Intervention | 52.4 (11.1) | 0.6 (-1.1,2.3) | 0.51 | -1.7 (-3.6, 0.3) | 0.09 | -1.1 (-3.8, 1.5) | 0.40 |
| WHO-5 |  |  |  |  |  |  |  |
| Control | 69.4 (14.8) | -2.6 (-6.7, 1.5) | 0.22 | -2.3 (-6.4, 1.8) | 0.28 | -3,8 (-7.9, 0.4) | 0.07 |
| Intervention | 70.6 (16.5) | -5.7 (-11.2, -0.2) | 0.04 | -6.1 (-11.6, -0.7) | 0.03 | -0.8 (-6.3, 4.7) | 0.78 |
| WHO-Overall QOL |  |  |  |  |  |  |  |
| Control | 3.9 (0.8) | -0.0 (-0.2, 0.2) | 0.92 | -0.0 (-0.2, 0.2) | 0.89 | -0.0 (-0.2, 0.2) | 0.93 |
| Intervention | 4.0 (0.7) | -0.2 (-0.5, 0.0) | 0.08 | -0.1 (-0.3, 0.2) | 0.49 | -0.1 (-0.3, 0.2) | 0.62 |
| PAID-5 |  |  |  |  |  |  |  |
| Control | 4.6 (4.2) | 0.8 (-0.2, 1.9) | 0.12 | 0.3 (-0.7, 1.3) | 0.55 | 0.6 (-0.4, 1.6) | 0.24 |
| Intervention | 3.9 (3.8) | 0.9 (-0.2, 2,0) | 0.12 | 0.7 (-0.5, 1.8) | 0.25 | 1.1 (-0.2, 2.4) | 0.09 |
| EQ-5D-5L |  |  |  |  |  |  |  |
| Control | 0.9 (0.1) | -0.0 (-0.1, 0.0) | 0.51 | -0.0 (-0.1, 0.0) | 0.07 | -0.0 (-0.1, 0.0) | 0.38 |
| Intervention | 0.8 (0.2) | 0.0 (-0.0, 0.0) | 0.35 | 0.0 (-0.0, 0.1) | 0.35 | 0.0 (-0.0, 0.1) | 0.40 |
| EQ-5D-VAS |  |  |  |  |  |  |  |
| Control | 70.6 (20.0) | -0.6 (-5.5, 4.4) | 0.82 | 2.1 (-2.9, 7.1) | 0.40 | 1.0 (-3.9, 5.9) | 0.69 |
| Intervention | 72.3 (19.9) | -1.7 (-7.5, 4.1) | 0.56 | -1.3 (-6.7, 4.1) | 0.63 | -1.4 (-6.8, 3.9) | 0.60 |
| WHO-Overall Health |  |  |  |  |  |  |  |
| Control | 3.3 (0.8) | 0.1 (-0.1, 0.3) | 0.47 | 0.2 (-0.0, 0.4) | 0.12 | 0.1 (-0,1, 0.3) | 0.24 |
| Intervention | 3.4 (0.8) | -0.0 (-0.3, 0.2) | 0.79 | 0.1 (-0.1, 0.3) | 0.48 | 0.1 (-0.2, 0.3) | 0.62 |
